# Food intolerance/malabsorption in Hashimoto’s thyroiditis

**DOI:** 10.1016/j.clinsp.2026.101168

**Published:** 2026-10-15

**Authors:** Wolfgang J. Schnedl, Simon Michaelis, Michael Schenk, Dietmar Enko, Sandra J. Holasek

**Affiliations:** a Division of Immunology, Otto Loewi Research Center, Medical University of Graz, Graz, Austria; b General Internal Medicine Practice, Bruck, Austria; c Institute of Clinical Chemistry and Laboratory Medicine, Hospital Hochsteiermark, Leoben, Austria; d Das Kinderwunsch Institut Schenk GmbH, Dobl, Austria; e Clinical Institute of Medical and Chemical Laboratory Diagnosis, Medical University of Graz, Graz, Austria

**Keywords:** Hashimoto's thyroiditis, Thyroid peroxidase, Fructose, Histamine, Lactose intolerance

## Abstract

**Context:**

The clinical significance of the interaction between the gastrointestinal system and the thyroid gland remains unclear. Moreover, a correlation between food intolerance/malabsorption, and thyroid disease, including Hashimoto's thyroiditis, awaits elucidation.

**Objectives:**

This study set out with the objective of identifying food intolerance/malabsorption, including Fructose Malabsorption (FM), Histamine Intolerance (HIT), and Lactose Intolerance (LIT), in patients presenting with abdominal symptoms within the Irritable Bowel Syndrome (IBS) spectrum who had also Hashimoto's thyroiditis.

**Methods:**

In this retrospective study, 221 patients with Hashimoto’s thyroiditis, defined by elevated Thyroid Peroxidase Antibodies (TPO-Ab), were included. A subgroup of 50 abdominal asymptomatic Hashimoto’s patients served as controls. Among 171 patients with abdominal symptoms FM and LIT were diagnosed using hydrogen breath tests, while HIT was defined by reduced serum diamine oxidase levels in combination with clinical response to a histamine-reduced diet.

**Results:**

Twenty-two were identified with FM, 40 with HIT and 42 with LIT; 19 had both LIT and FM, 31 had LIT and HIT, and 17 had LIT, FM, and HIT. However, there were no statistically significant differences in TSH or TPO-Ab levels between patients with FM, HIT, LIT, or their combinations (p = 0.739 and p = 0.460, respectively). Thyroid function and TPO-Ab levels were similar between symptomatic and asymptomatic patients.

**Conclusion:**

In summary, this study assumes that food intolerance/malabsorption, including FM, HIT, and LIT, may coexist in patients with Hashimoto's thyroiditis who present with IBS-spectrum abdominal symptoms.

## Highlights

• Food intolerance/malabsorption and thyroid disorders remain poorly understood.
• Patients with Hashimoto’s thyroiditis present with IBS-spectrum abdominal symptoms.
• Fructose malabsorption, lactose and histamine intolerance may co-occur.
• These conditions may occur alone or in combination with Hashimoto’s thyroiditis.

## Introduction

Hashimoto’s thyroiditis, also known as chronic lymphocytic thyroiditis, is the most prevalent autoimmune disease worldwide. In this condition, Thyroid Peroxidase (TPO) serves as the primary autoantigen, and serum TPO-Antibody (TPO-Ab) levels correlate with the extent of lymphocytic infiltration in thyroid tissue.^1^ Therefore, measurement of TPO-Ab titers plays a central role in the diagnosis and clinical evaluation of Hashimoto’s thyroiditis.^2^ In routine clinical practice, serum Thyroid-Stimulating Hormone (TSH) remains the primary laboratory parameter for assessing thyroid function.^3^

The Gastrointestinal (GI) tract is increasingly recognized as a target of thyroid hormones, a concept often referred to as the gut-thyroid axis.^4^ This bidirectional interaction may cause the frequent overlap between thyroid-related symptoms and GI complaints. It has been suggested that certain foods and GI conditions can influence the absorption of Levothyroxine (LT-4) in patients with thyroid disorders.^5^ In individuals with Hashimoto’s thyroiditis, studies indicate a correlation between food intolerances ‒ particularly Lactose Intolerance (LIT) ‒ and impaired intestinal LT-4 absorption.^6^ Clinically, there is considerable symptom overlap between food intolerance/malabsorption and symptoms within the Irritable Bowel Syndrome (IBS) spectrum.^7^ Consequently, a personalized and comprehensive diagnostic approach is recommended when evaluating suspected conditions such as Fructose Malabsorption (FM), Histamine Intolerance (HIT), LIT, and *Helicobacter pylori* (*H. pylori*) infection in patients presenting with symptoms of the IBS spectrum.^8^ The present study sought to ascertain whether food intolerance/malabsorption, including FM, HIT and LIT, are responsible for symptoms within the IBS spectrum in patients who also have Hashimoto's thyroiditis.

## Material and methods

### Participants and study design

This retrospective study evaluated 301 consecutive white patients with elevated Thyroid Peroxidase Antibodies (TPO-Ab) and Thyrotropin Receptor Antibodies (TSH-R-Ab) within normal range, consistent with a diagnosis of Hashimoto's disease. A total of 221 patients were included in the study after excluding 80 patients (26.6%) with *H. pylori* infection. All patients were treated with Levothyroxine (LT-4) tablets. Data were retrieved from institutional archives covering the period between December 2016 and January 2023.

Medical records of patients presenting with GI symptoms within the spectrum of Irritable Bowel Syndrome (IBS) suggestive of food intolerance/malabsorption were retrospectively reviewed. Reported symptoms included abdominal pain, bloating, flatulence, loose stools, diarrhea, postprandial fullness, and/or constipation. Abdominal symptomatic 171 Hashimoto’s disease patients underwent diagnostic evaluation for food intolerance/malabsorption according to our standard clinical practice. The control group consisted of 50 patients with Hashimoto's disease who did not report GI symptoms and, consequently, did not undergo further evaluation for food intolerance or malabsorption. The anthropometric characteristics of the subjects in this study, in addition to their daily requirements of thyroxine, could not be ascertained. Participants who declined to undergo all study procedures or had TSH Receptor Antibody (TSH-R-Ab) levels above the normal range, indicative of Basedow's or Graves' disease, were excluded from the study. Additional exclusion criteria included thyroid cancer; concomitant autoimmune diseases, including celiac disease, autoimmune gastritis, rheumatoid arthritis, and type 1 diabetes mellitus; food intolerance or malabsorption in combination with *H. pylori* gastritis; inflammatory bowel disease; recent antibiotic therapy; prior gastrointestinal surgery; and evidence of systemic or intestinal infection or inflammation. Furthermore, screening colonoscopy was performed in participants aged > 50-years. To avoid potential interference with the Hydrogen (H_2_) breath tests, colonoscopy was not performed within four weeks of breath testing. It should be noted that endoscopy with biopsy and histopathological examination of the GI mucosa and radiological imaging, including ultrasonography, are not interchangeable diagnostic modalities for excluding organic GI diseases.

### Laboratory evaluations

Venous blood samples were collected after an overnight fast of at least 12 h, with Levothyroxine (LT-4) withheld until after blood collection. Samples were obtained at the time of patient presentation, preceding any evaluation of their dietary habits or adherence to a specific diet. The diagnosis of Histamine Intolerance (HIT) was established based on a serum Diamine Oxidase (DAO) level of less than 10 U/mL in conjunction with documented clinical improvement following adherence to a histamine-reduced diet. Serum DAO concentrations were measured using the radio extraction assay DAO Rea 100 (Sciotec Diagnostic Technologies, Tulln, Austria).

Serum Thyrotropin (TSH) levels were measured using a chemiluminescence immunoassay on the Atellica IM Analyzer (Siemens Healthcare GmbH, Erlangen, Germany). TSH levels below 3.8 U/mL were found to be within the normal range. Diagnostic thyroid peroxidase antibodies (anti-TPO) with values more than 5 U/mL were considered indicative of pathological elevation. TSH receptor antibodies (TSH-R-Ab) were assessed using a chemiluminescence assay performed on the Alinity I Immunology Analyzer (Abbott, Chicago, IL, USA). Serum TSH-R-Ab values below 3.09 U/mL were considered to be within the normal range. IgA antibodies against tissue transglutaminase (anti-tTG IgA) were measured using a commercially available Enzyme-Linked Immunosorbent Assay (ELISA) (Euro Diagnostica AB, Malmö, Sweden). Anti-tTG IgA levels < 10 U/mL were considered within the normal range, whereas values ≥10 U/mL were classified as elevated. *H. pylori* infection was assessed either by ELISA-based serological testing (Serion, Würzburg, Germany) for screening or by histological examination of gastric mucosal biopsy specimens obtained during upper GI endoscopy. In the cohort of patients evaluated, a histologic evaluation of the gastric mucosa was performed on patients' post-eradication or with specific gastric pain.

### Hydrogen breath tests

H₂ breath testing was subsequently performed using the Gastrolyzer device (Bedfont Scientific Ltd., Kent, UK). The H₂ breath test was used to evaluate the presence of LIT. For the lactose H_2_ test, participants ingested 50g of lactose dissolved in 200 mL of water. Blood glucose levels were measured under fasting conditions using the glucose oxidase method and were reassessed at 60- and 120 min after lactose ingestion. LIT was diagnosed if expiratory H_2_ increased by more than 20 ppm above baseline and/or if the rise in blood glucose was less than 20 mg/dL compared with the fasting value. Two weeks after the lactose breath test, a H_2_ breath test was performed to assess FM. After a 12 h overnight fast, during which only plain water was permitted, participants ingested 25g of fructose dissolved in 200 mL of water. Expiratory H_2_ levels were measured over a 120 min period. An increase of more than 20 ppm above baseline was considered diagnostic of FM. Prior to testing, participants followed a preparatory diet excluding poorly absorbable, fermentable carbohydrates and fiber. They were instructed to avoid antibiotics, laxatives, dietary supplements, and probiotics before the test. Additionally, smoking, chewing gum, and strenuous physical activity were prohibited for at least four hours prior to the procedure. Compliance with these instructions was confirmed before initiating the breath test.

### Statistical analysis

Statistical analyses were performed with IBM SPSS statistics version 28.0 (IBM, Armonk, NY, USA), and GraphPad Pism version 10.3.1. (GraphPad Software, San Diego, CA, USA) was used for the generation of figures. As a nonparametric statistical test for paired comparisons of TSH and TPO-Ab measurements, the Kruskal-Wallis test and Dunn's post hoc test with Bonferroni correction was used. Statistical significance was defined as a p-value of less than 0.05.

## Results

During the retrospective evaluation of 221 patients with Hashimoto's disease, patients were divided into groups based on the presence or absence of abdominal symptoms within the IBS spectrum. The group without abdominal symptoms included 50 patients (3 men and 47 women), with a median age of 50-years (range: 23.7–77.9 years). The evaluation in this control group did not include H_2_ breath tests, DAO, anti-tTG IgA values and *H. pylori* testing.

As illustrated in Table 1, the study examined a total of 171 patients who presented with abdominal symptoms of the IBS spectrum. Twenty-two subjects were diagnosed with Hashimoto's disease and concomitant FM, and the H_2_ breath test did not indicate LIT. Furthermore, DAO values were found to be normal (> 10 U/mL). Conversely, 42 patients diagnosed with Hashimoto's disease were found to have LIT, while FM was not detected by the FM hydrogen breath tests and DAO values were normal > 10 U/mL. Furthermore, in patients diagnosed with Hashimoto's disease and HIT, as indicated by DAO values of less than 10 U/mL, the H_2_ breath test did not reveal the presence of LIT or FM. In addition, in patients diagnosed with Hashimoto's disease and LIT who also had concomitant FM, DAO levels were found to be > 10 U/mL. Patients diagnosed with Hashimoto's disease and LIT in combination with HIT, as indicated by DAO values < 10 U/mL, were also included in the study. However, the H_2_ breath tests did not detect FM in this group.

**Table 1:** Food intolerance/malabsorption and combinations thereof found in 171 Hashimoto patients with symptoms of the IBS spectrum.

Food intolerance/ Malabsorption	Number of patients	Percent (%)	Male/ Female	Median age in years (min‒max)	Median diamine oxidase (U/mL)
LIT	42	24.6	4/38	53.3 (23.9‒78.8)	16.5 (10.7‒70.5)
FM	22	12.9	2/20	48.1 (25.8‒75.3)	18.5 (10.3‒45.6)
HIT	40	23.4	3/37	47.1 (18‒78.8)	6.6 (1.5‒9.9)
LIT + FM	19	11.1	3/16	52.2 (23‒71.8)	16.7 (10.2‒31.4)
LIT + HIT	31	18.1	1/30	38.9 (21.3‒83.5)	6.7 (1.5‒9.7)
LIT + FM + HIT	17	9.9	2/15	55.8 (23.3‒76.7)	6 (1.5‒9.5)

LIT, Lactose Intolerance; FM, Fructose Malabsorption; HIT, Histamine Intolerance.

To exclude the possibility of Basedow’s and Graves’ disease, TSH Receptor Antibody (TSH-R-Ab) levels were measured in all patients and found to be within the normal range. Additionally, to rule out celiac disease, none of the 171 patients showed anti-tTG IgA. Furthermore, signs of *H. pylori* infection were not detected in any of the 171 patients included in the study. All patients over the age of 50 underwent a colonoscopy, which revealed no pathology. However, it is important to note that these procedures were not conducted within a period of four weeks prior to breath tests. None of the patients exhibited signs of infection.

Using the Kruskal-Wallis test for comparison of all Hashimoto groups between the data for TSH and TPO-Ab did not reveal any statistical differences, p = 0.739 and p = 0.46, respectively (Fig. 1, Fig. 2, Fig. 3, Fig. 4). A comparison of the groups of patients revealed no statistically significant differences in terms of age (p = 0.451). Subsequent to the Kruskal-Wallis test, Dunn's post hoc test with Bonferroni correction was performed for multiple comparisons. Following statistical analyses did not reveal any statistically significant differences between the various subgroups.

**Fig. 1:**
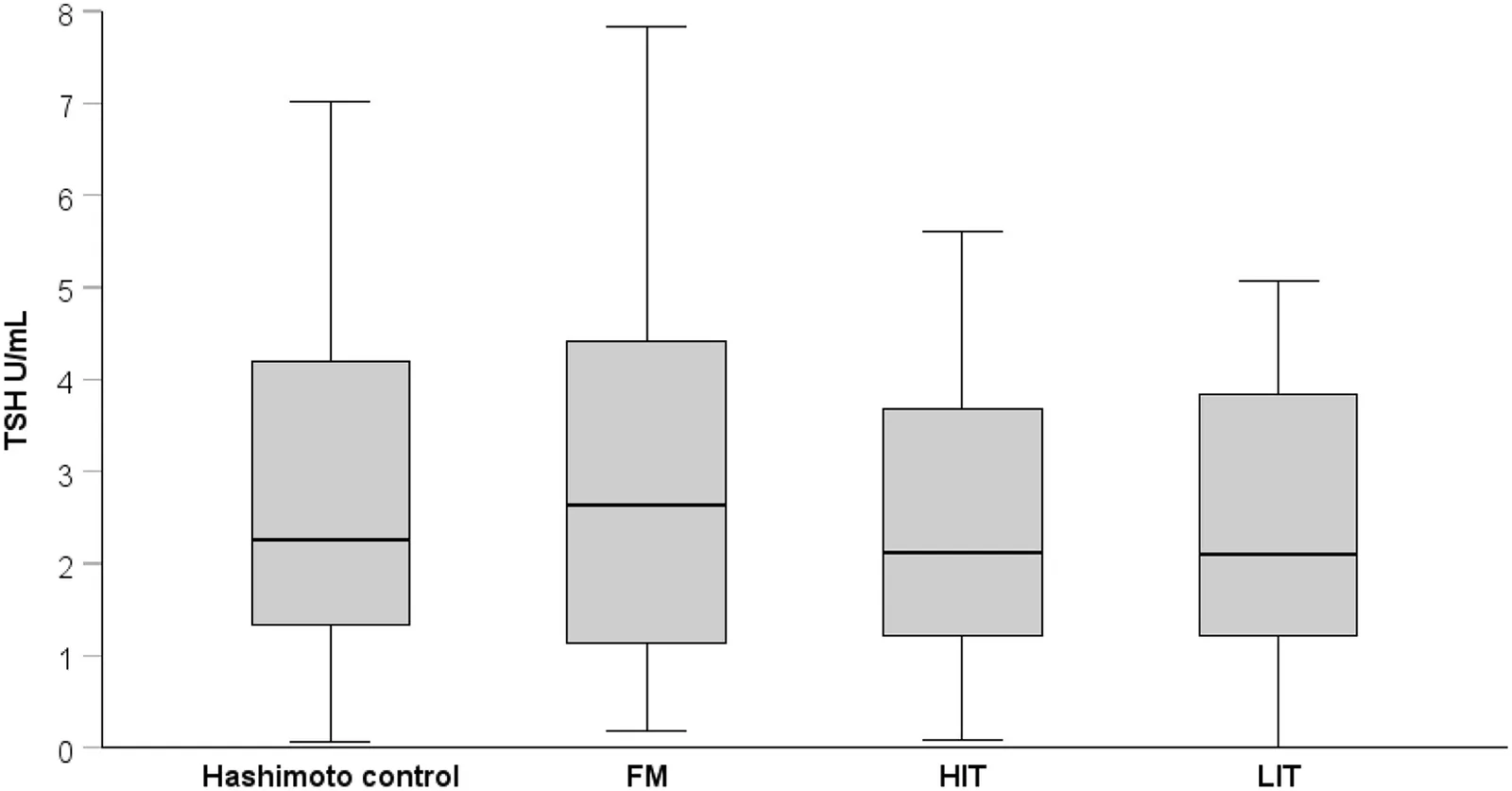
The boxplot of TSH measurements in the control group of Hashimoto's disease, as well as in the FM, LIT and HIT groups, each of which had Hashimoto's thyroiditis, demonstrated no statistically significant difference (p = 0.7). FM, Fructose malabsorption; HIT, Histamine Intolerance; LIT, Lactose intolerance; TSH, Thyrotropin.

**Fig. 2:**
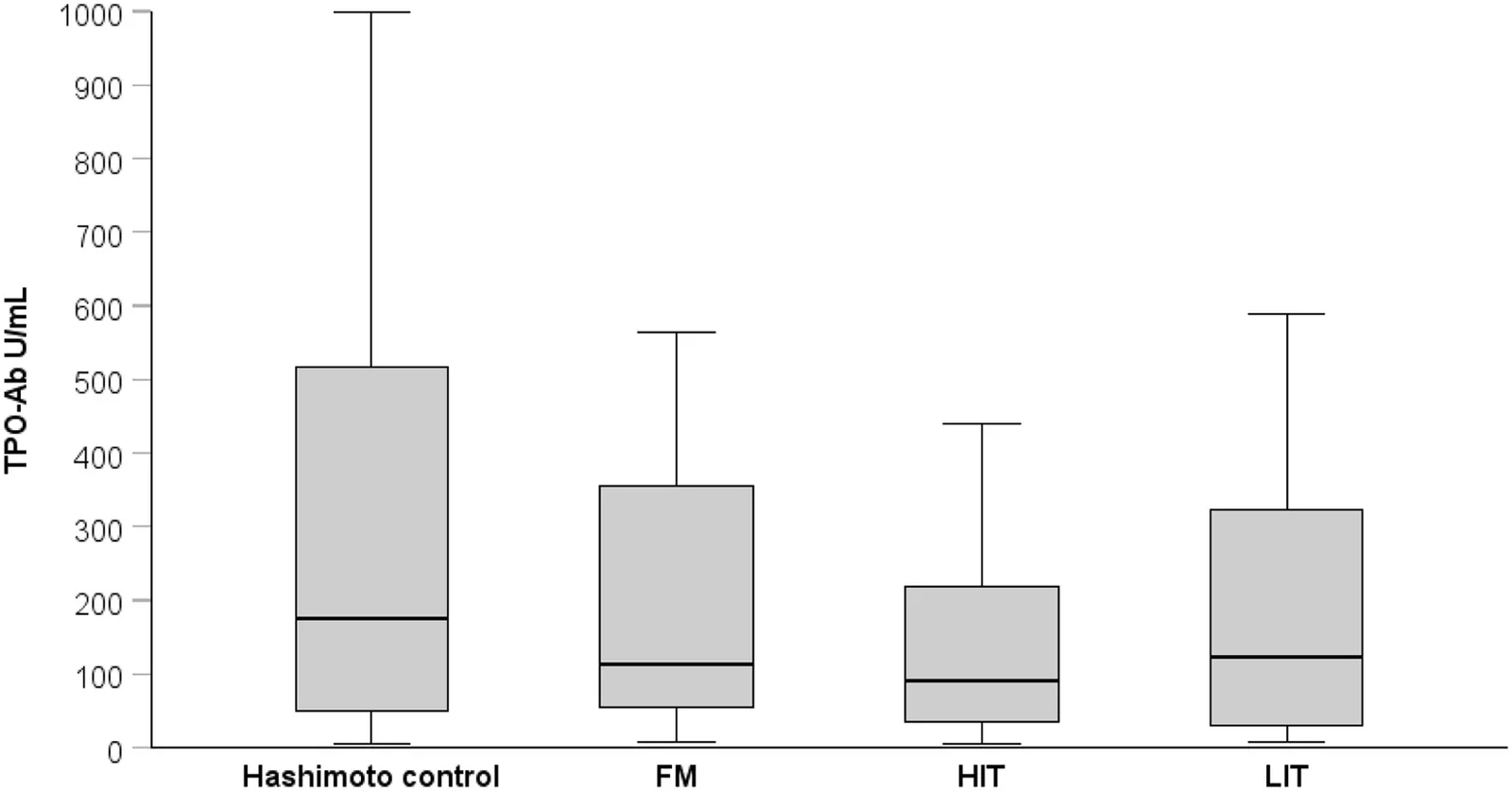
The boxplot of TPO-Ab values showed no statistically significant differences between the control group with Hashimoto’s disease and the FM, LIT, and HIT groups, all of which had Hashimoto’s thyroiditis (p = 0.272). FM, Fructose malabsorption; HIT, Histamine Intolerance; LIT, Lactose Intolerance; TPO-Ab, Thyroid peroxidase antibodies.

**Fig. 3:**
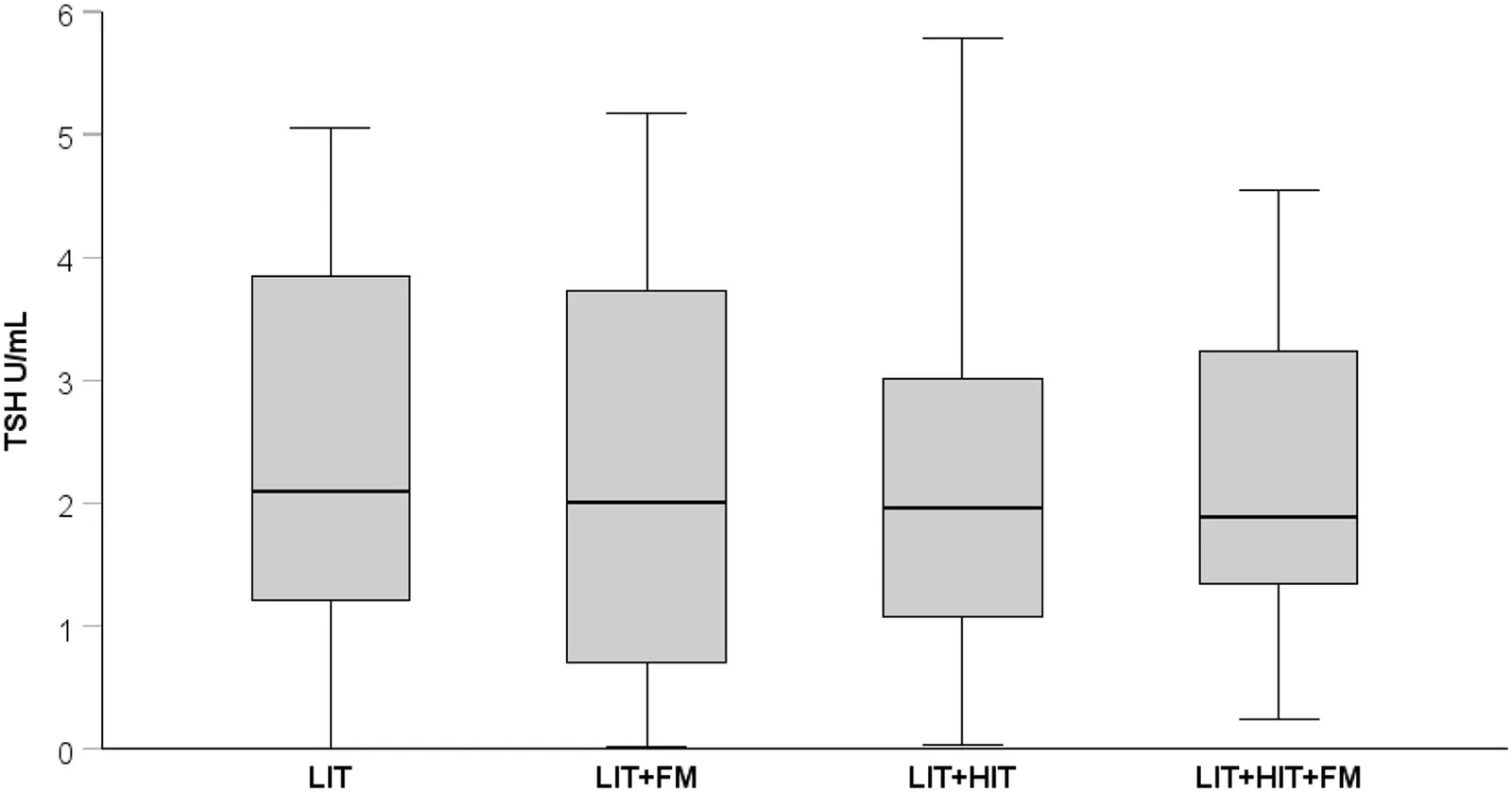
The boxplot analysis of TSH concentrations demonstrated no statistically significant differences between the LIT group with Hashimoto’s disease and the LIT + FM, LIT + HIT and LIT + HIT + FM groups, each of which had confirmed Hashimoto’s thyroiditis (p = 0.861). FM, Fructose malabsorption; HIT, Histamine Intolerance; LIT, Lactose Intolerance; TSH, Thyrotropin.

**Fig. 4:**
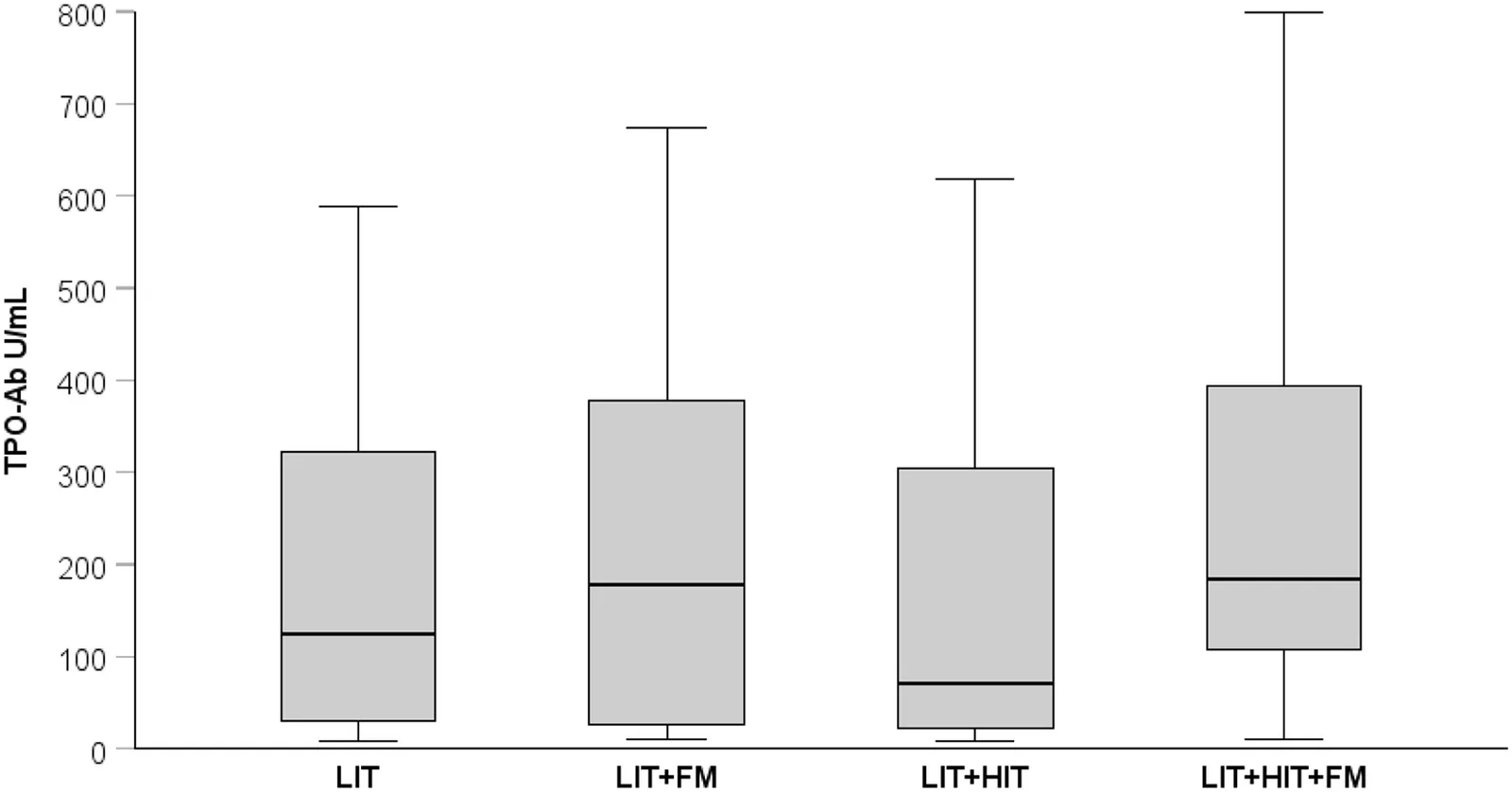
The boxplot of TPO-Ab values revealed no statistically significant differences when comparing the LIT group with Hashimoto’s disease to the LIT + FM, LIT + HIT and LIT + HIT + FM groups, all of which had Hashimoto’s thyroiditis (p = 0.569). FM, Fructose Malabsorption; HIT, Histamine Intolerance; LIT, Lactose Intolerance; TPO-Ab, Thyroid peroxidase antibodies.

## Discussion

Research has identified the gut microbiota as a key factor influencing both general health and disease, including the influence on Hashimoto's thyroid disease.^9^ The hypothesis that the gut microbiota plays a role in autoimmune thyroid diseases is supported by observational and interventional studies in animal models. This has been confirmed in some reports from human cohorts.^10^ There is an increasing scientific interest in the relationship between the GI tract and the thyroid gland. Research has identified the GI tract as a target of thyroid hormones, and there is growing evidence to suggest a link between thyroid disorders and gut conditions.^4^ The studies presented thus far suggest a correlation between food intolerance, principally LIT, and alterations in LT-4 absorption in thyroid disease.^11^ Here, we suggest that patients with symptoms within the IBS spectrum and a diagnosis of Hashimoto’s thyroiditis may also have coexisting food intolerance/malabsorption, including FM, HIT and LIT.

Despite the lack of clear evidence supporting specific dietary recommendations for patients with Hashimoto’s thyroiditis.^12^ the potential role of nutritional deficiencies continues to be discussed. In particular, nutrients such as iodine, selenium, magnesium, iron, vitamin B12, and vitamin D remain of interest in this context. The possible benefits of an anti-inflammatory diet have also been proposed.^13^ Such a diet, typically characterized by a high intake of vitamins and minerals and a lower consumption of animal products, may offer some anti-inflammatory effects. At present, there is insufficient evidence from research to support the recommendation of these dietary strategies, or the recently popular gluten-free diet, for all individuals with Hashimoto's thyroiditis.^14^ Food intolerance/malabsorption - including FM, HIT, and LIT - present with symptoms that overlap with those of the IBS spectrum.^7^ These symptoms are likely influenced by multiple interacting factors, such as the type of sugars or proteins consumed, the fermentative capacity of the gut microbiota, individual sensitivity to gases and fermentation products, the colon’s ability to absorb water, meal osmolality, dietary fat content, and gastric emptying time.^15^ From a pathophysiological perspective, mechanisms may include variations in mucosal glucose transporter expression in FM, as well as intestinal enzyme deficiencies in conditions such as LIT or HIT. Although it has been proposed that chronic inflammation in patients with Hashimoto’s thyroiditis could influence histamine metabolism.^16^ This was not supported by the data presented here. In addition, our findings indicate that TSH and TPO-Ab concentrations in patients with HIT are comparable regardless of the presence or absence of food intolerance or malabsorption. This observation further supports the hypothesis that these represent two coexisting conditions rather than one directly influencing the other.

One study has suggested that patients with Hashimoto’s thyroiditis may be more likely to develop food intolerances compared to the general healthy population, based on the detection of food-specific IgG antibodies.^17^ However, IgG antibodies are not considered a reliable diagnostic tool for non-allergic food intolerance/malabsorption. In contrast, the European consensus-based clinical practice guideline for H₂ breath testing, which was the basis of our research, recommends the use of these breath tests for the diagnosis of conditions such as FM and LIT.^18^ Yet, further clinical and experimental studies are required to elucidate the impact of food intolerance/malabsorption on thyroid diseases. As an observational study, the STROBE Statement was followed. The limitations of this study include its retrospective design, the potential for selection bias as it was conducted at a single center, and the limited ethnic diversity of the patient cohort. Due to the retrospective study design, the sample size was determined by the number of eligible patients identified from the archived records. Consequently, some subgroup analyses included only a small number of patients, resulting in limited statistical power.

Conventional LT-4 tablets contain lactose as an excipient; however, the exact amount is typically not disclosed by manufacturers. Most individuals with LIT are able to tolerate up to approximately 12g of lactose in a single dose with minimal or no symptoms, although individual sensitivity can vary considerably. Some more sensitive individuals may experience symptoms with amounts below 6g.^19^ According to the National Health Service Specialist Pharmacy Service, daily lactose exposure from medications rarely exceeds 2g per day, even when multiple tablets are taken.^20^ Nevertheless, lactose-free LT-4 formulations have been developed, which are generally more expensive. These alternatives have been proposed as a potential option to improve absorption in patients with LIT and may contribute to normalization of TSH levels.^21^ No notable differences in TSH levels were observed among patients receiving LT-4 therapy, irrespective of whether they were in the control group or had FM, HIT, or LIT.

Patients treated with LT-4 may also have coexisting conditions that could reduce its GI absorption.^6^ In particular, disorders affecting the gastric or small intestinal epithelium may influence LT-4 absorption. These include conditions such as ulcerative colitis, celiac disease, prior bariatric surgery, *H. pylori* infection, food intolerance, and gastritis, among others, each with distinct underlying mechanisms. LIT as food intolerance, which is often mentioned in this context, is primarily caused by a genetic deficiency of the enzyme lactase, leading to impaired digestion of lactose found in milk and dairy products. However, despite its widespread use and status as one of the most commonly prescribed medications worldwide, further research is needed to fully elucidate its effects.^22^ The hypothesis that LIT significantly impairs LT-4 absorption is based on relatively limited evidence, including small patient cohorts and a modest number of reported cases.^5^^,^^6^^,^^23^, ^24^, ^25^ In contrast, it has recently been reported that LIT does not appear to affect the required dose of LT-4 in patients with Hashimoto’s disease. Accordingly, LIT has been considered a relatively minor factor in LT-4 malabsorption.^25^ Decreased TSH and TPO-Ab values have been reported in Hashimoto's patients with compliant intake of LT-4 tablets.^26^ The observations reported here demonstrate that there seem no meaningful differences in TSH or diagnostic TPO-Ab levels between patients with Hashimoto's thyroiditis and LIT, those with Hashimoto's disease alone, or in comparison with patients with Hashimoto's thyroiditis and HIT or FM (Fig. 1, Fig. 2).

However, a more extensive review of the available data suggests the presence of some inconsistency.^6^ It remains unclear whether thyroid function can be uniformly defined as normal, given inter- and intra-individual variability in thyroid parameters, as well as transient fluctuations, age-related differences, and ethnic variation. Moreover, concerns regarding noncompliance are prevalent and ought to be duly considered.^26^ Consequently, the most effective approach to assess thyroid function is to do so on an individual basis, taking into account each patient's unique clinical presentation.^27^ In particular, the prevalence of LIT in the global population is high at approximately 70%, and LIT prevalence was found to be 75% in patients diagnosed with Hashimoto's disease.^11^ It is acknowledged that the presence of coexisting food intolerance or malabsorption conditions may have an impact on one another's exhaled H₂ values during H_2_ breath testing, thereby suggesting alterations in digestive processes.^28^^,^^29^ Nevertheless, no correlation was identified between food intolerance/malabsorption and TSH or TPO-Ab levels in patients with Hashimoto's thyroiditis (Fig. 3, Fig. 4).

Autoimmune thyroid disease, including Hashimoto's thyroiditis, frequently coexists with other organ-specific and systemic autoimmune disorders.^30^ Nonetheless, a plethora of associations have been delineated between autoimmune markers of Hashimoto's thyroiditis and extra-thyroidal diseases, encompassing neurological, cardiovascular, musculoskeletal, and hematological manifestations.^31^ Thyroid hormones play a key role in skeletal development and growth, as well as in maintaining adult bone mass and strength.^32^ Although no connection of cartilage thickness with Hashimoto's thyroiditis was recognized.^33^ In individuals presenting with abdominal symptoms within the IBS spectrum and a diagnosis of Hashimoto's thyroiditis, further evaluation for potential food intolerance/malabsorption, including FM, HIT, and LIT, may be indicated. In the event of an individual being diagnosed with food intolerance or malabsorption, it is recommended that they be referred to a registered dietitian who possesses expertise in these conditions. The purpose of this referral is to develop a personalized dietary plan. A proposed plan for the management of this condition would entail the targeted reduction and/or elimination of foods that precipitate symptoms, with this process being guided by a systematic assessment and patient-reported outcomes. It is imperative that dietary adjustments are tailored to each patient's individual tolerance levels in order to optimize long-term symptom management while avoiding unnecessary restrictions.

## Conclusion

In summary, the present study assumes that food intolerance/malabsorption disorders, including FM, HIT and LIT, may co-occur in patients with Hashimoto's thyroiditis who experience IBS-spectrum abdominal symptoms.

## Ethics statement

The Ethical Committee of the Johannes Kepler University in Linz, Austria (n° K-107-16) approved the review of existing database records. All procedures were conducted in accordance with the ethical principles outlined in the Declaration of Helsinki. Informed consent was obtained from all human subjects involved in the study.

## AI-Assisted language editing statement

The authors used ChatGPT (OpenAI) solely to improve the English language, grammar, spelling, and readability of the manuscript.

## Author’s contribution

Wolfgang J. Schnedl carried out the study design, patient recruitment, data collection, data analysis, discussion of results, and manuscript drafting. Simon Michaelis and Michael Schenk were responsible for patient recruitment, laboratory data determination, data analysis, manuscript drafting, and discussion of results. Dietmar Enko was involved in data analysis, study design, laboratory data determination, and manuscript writing. Sandra J. Holasek contributed to the study design, data analysis, manuscript writing, and discussion of results. All authors reviewed and approve the final manuscript.

## Funding

Open access funding provided by the 10.13039/501100010109Medical University of Graz. This study did not receive financial support from any commercial or nonprofit funding bodies.

## Conflicts of interest

Wolfgang J. Schnedl has received speaker honoraria from Sciotec. The remaining authors report no conflicts of interest.

## Data Availability

The datasets used and/or analyzed during the current study are available from the corresponding author on reasonable request.
